# A case of catastrophic epistaxis from the internal carotid artery due to multiple surgeries and irradiations for pituitary tumor: Rescued utilizing high‐flow bypass and simultaneous skull base reconstruction

**DOI:** 10.1002/ccr3.4697

**Published:** 2021-08-25

**Authors:** Kosuke Takabayashi, Seiji Takebayashi, Juro Sakurai, Shuho Gotoh, Katsumi Takizawa

**Affiliations:** ^1^ Department of Otorhinolaryngology Japanese Red Cross Asahikawa Hospital Hokkaido Japan; ^2^ Department of Neurosurgery Japanese Red Cross Asahikawa Hospital Hokkaido Japan

**Keywords:** epistaxis, internal carotid artery, irradiation, rupture, vascular surgical procedures

## Abstract

Revascularization for internal carotid artery rupture should be considered immediately under the situation where endovascular treatment is not indicated. Revascularization can prevent the risk of hemorrhage during skull base reconstruction.

## Graphical abstract

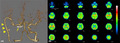

## 1 INTRODUCTION

A patient with internal carotid artery (ICA) rupture due to multiple irradiations underwent revascularization with high‐flow bypass under the condition that endovascular treatment could not be performed. It was possible to safely remove necrotic tissues and reconstruct the skull base using trapping of the ruptured ICA.

Massive epistaxis caused by rupture of the internal carotid artery (ICA) is a crisis. Failure of hemostasis or in controlling treatment complications has led to an increased mortality rate by one‐third.^1^, ^2^ For decades, previous studies have reported treatment methods, such as high‐flow bypass^1^, ^3^, ^4^, ^5^ and endovascular treatment.^1^, ^2^, ^6^, ^7^, ^8^, ^9^, ^10^, ^11^ In addition, it has been reported that one of the causes of ICA rupture was skull base osteonecrosis due to second course of irradiation for nasopharyngeal carcinoma (NPC)^1^, ^2^, ^6^, ^8^, ^9^, ^10^ or skull base tumor.^7^

Although several studies have reported that endovascular treatment should be considered first,^1^, ^2^, ^6^, ^7^, ^8^, ^9^, ^10^, ^11^ complete revascularization rather than endovascular treatment should be preferred in some situations: The balloon occlusion test (BOT) is not reliable due to coma and cannot be tolerated by some patients.^1^, ^2^, ^6^, ^7^ Meanwhile, most of the reported cases were cases of recurrent malignant tumors.^1^, ^2^, ^6^, ^8^, ^9^, ^10^

Herein, we describe a case of ICA rupture requiring radical revisualization and skull base reconstruction. In this case, neither a ruptured site nor pseudoaneurysm could be identified. Moreover, second course of irradiation‐induced osteonecrosis was due to the primary disease: pituitary adenoma. This report aimed to present how we chose procedures for this atypical condition, in which the prerequisites for endovascular treatment could not be identified, and long‐term prognosis could be improved after definitive treatment was achieved.

## 2 CASE REPORT

**FIGURE 1:**
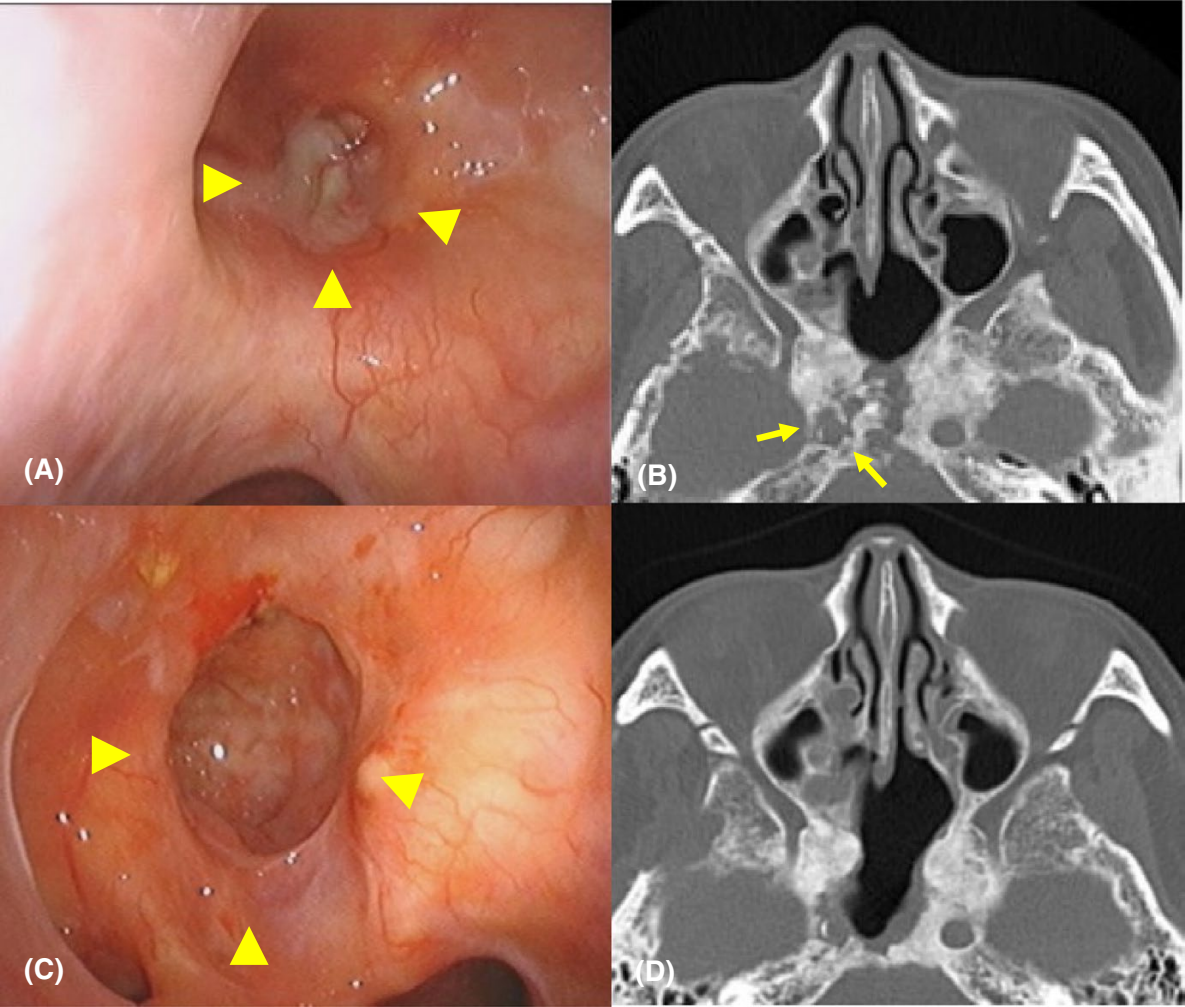
A. Preoperative nasal endoscopic finding indicating the discharge of pus from the infected sphenoid sinus, including osteonecrosis. Arrowheads indicate the sphenoid sinus. B. Preoperative axial computed tomography (CT) showing osteonecrosis of the sphenoid sinus, with damage to the right internal carotid canal. Arrows indicate the right internal carotid canal. C. Postoperative nasal endoscopic findings 15 months after surgery indicating the reconstructed sphenoid sinus with removal of necrotic tissue. Arrowheads indicate the reconstructed sphenoid sinus. D. Postoperative axial CT 15 months after surgery showing the reconstructed sphenoid sinus and skull base

**FIGURE 2:**
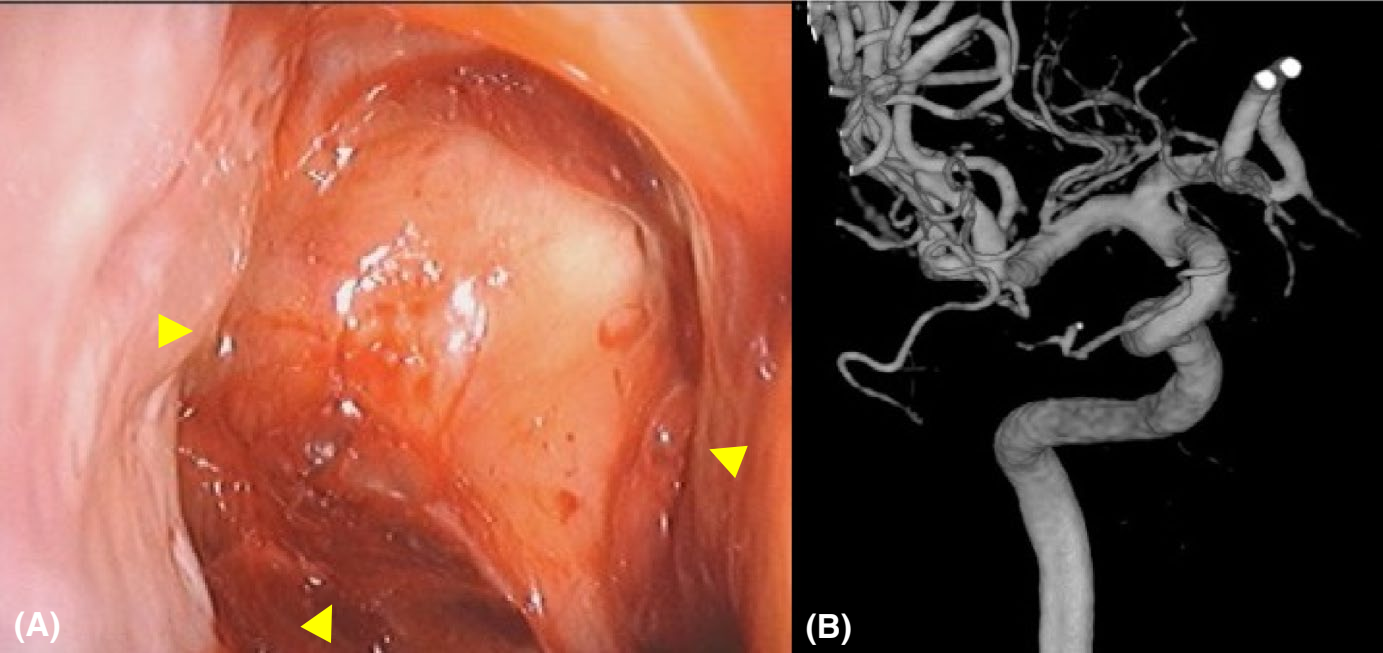
A. Endoscopic finding during spontaneous hemostasis indicating that the sphenoid sinus is filled with clots and flows into the nasal cavity. Arrowheads indicate the sphenoid sinus. B. The conventional angiography showing no hemorrhage site or pseudoaneurysm in the internal carotid artery

A 67‐year‐old woman, who has been followed up for osteonecrosis of the sphenoid bone, right blindness, and hypopituitarism by our department in the last 3 years after multidisciplinary treatment of a pituitary tumor 20 years ago, presented with massive epistaxis. She had undergone two transsphenoidal microsurgeries and craniotomy for pituitary adenoma, as well as radiotherapy, including cobalt brachytherapy and gamma knife. On the day before her presentation at our hospital, a brief loss of consciousness was observed due to a sudden massive epistaxis that fortunately stopped spontaneously. An otorhinolaryngologist, who had followed her up for osteonecrosis, especially the bone around the C3 segment of the right ICA that disappeared after chronic infection for 3 years (Figure 1A, B), considered a possibility of right ICA rupture due to her episode of massive epistaxis. Therefore, he consulted with neurosurgeons to evaluate the source of epistaxis. While waiting for the neurosurgeons, a massive epistaxis suddenly occurred again with loss of consciousness. Accordingly, the otorhinolaryngologist urgently attempted to stop bleeding using total nasal packing with a balloon catheter and impregnated ointment gauze. However, epistaxis continued despite the procedure and fortunately stopped spontaneously within a few minutes (Figure 2A). Although the neurosurgeons performed conventional angiography, no findings were noticeable in the right ICA (Figure 2B). Therefore, the neurosurgeons and otorhinolaryngologists first suggested that one of the peripheral branches of the right external carotid artery (ECA) was the source. Therefore, the neurosurgeon occluded the right sphenopalatine artery from which the contrast medium appeared to leak slightly. The patient was admitted to the neurosurgical department with a generous cooperation of the otorhinolaryngologist performing blood transfusion and strict follow‐up, in addition to suggesting ICA rupture.

After admission, massive epistaxis suddenly occurred again despite right sphenopalatine artery occlusion. Therefore, a high‐flow bypass between the second segment of the right middle cerebral artery (MCA) and the right cervical ECA was urgently performed. This procedure comprised using radial artery (RA) graft, surgical trapping of the right ICA, and skull base reconstruction with a pedicle mucosal flap harvested from the left nasal floor accompanied by removal of necrotic tissue. First, a RA graft was sutured to the second segment of the right MCA end‐to‐side by interrupted suture and the right cervical ECA end‐to‐side by continuous suture. The ophthalmic segment of the right ICA was ligated using a clip, and the cervical segment was ligated. Thereafter, the otorhinolaryngologist endoscopically removed necrotic tissues on the sphenoidal bone without any damage to the dura mater and then covered the skull base and sphenoid sinus with a vascular pedicled nasomucosal flap harvested from the left nasal floor.

**FIGURE 3:**
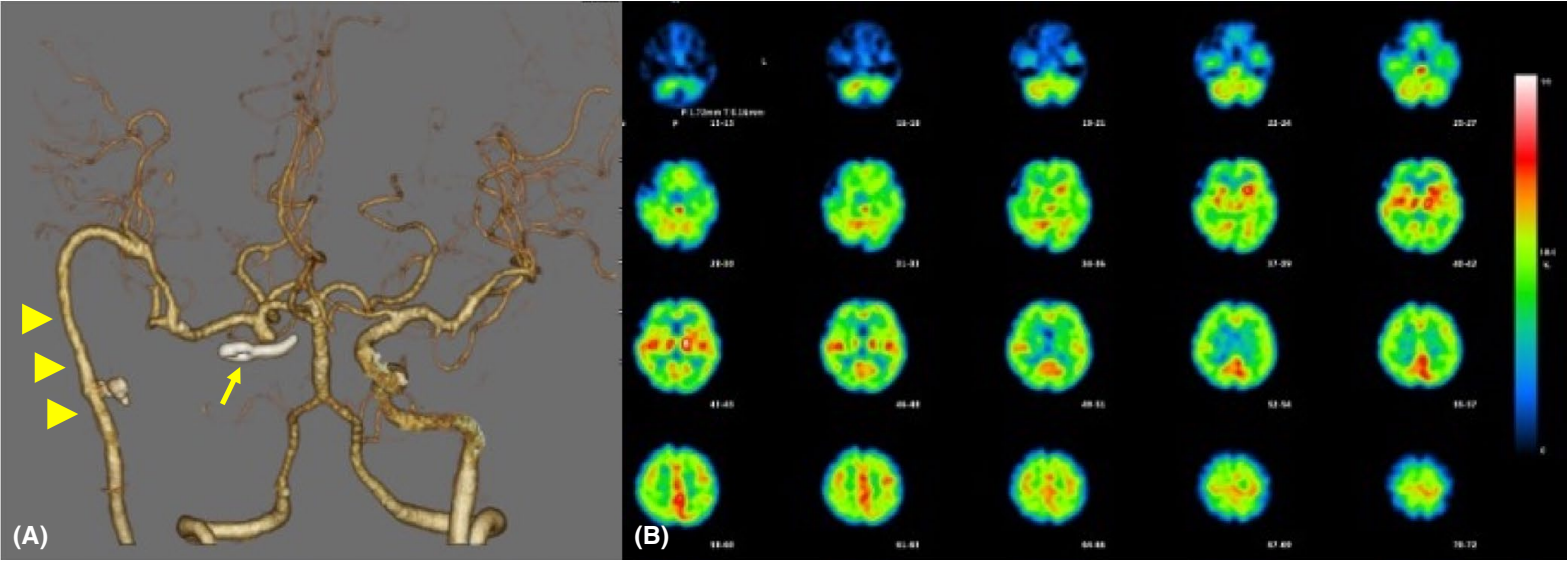
A. Postoperative CT angiography showing that revascularization is achieved by high‐flow bypass and peripheral arteries are sufficiently visualized. Arrowheads indicate radial artery graft as a high‐flow bypass. B. Postoperative single‐photon emission computed tomography image showing no decrease in cerebral blood flow

No postoperative epistaxis was observed. Although small watershed infarcts of the right hemisphere were observed, left paresis was transient after surgery. The patient was discharged 1 month after surgery without any residual disability. Revascularization was successful, and there was no evidence of decreased blood flow on single‐photon emission computed tomography (Figure 3A,B). Additionally, chronic infection of the sphenoid bone improved (Figure 1C,D). She has been uneventful for 15 months postoperatively.

## 3 DISCUSSION

In this case, we selected a combination of revascularization and skull base reconstruction. Previously reported ICA rupture cases due to osteonecrosis were almost recurrent NPC cases, and probably, no long‐term prognosis could be expected.^1^, ^2^, ^6^, ^8^, ^9^, ^10^ In contrast, the primary disease as pituitary adenoma had been stable for a long time. Therefore, complications after a long period of time, such as left ICA rupture, should be considered when necrotic tissue on the sphenoid bone was not removed. Hence, we considered that if complete revascularization had been established, skull base reconstruction with the removal of necrotic tissue would also be necessary to prevent complications after a long period of time.

Osteoradionecrosis, resulting from a second course of radiotherapy, affected the right ICA rupture, which might have been caused by obstruction of the vasa vasorum, premature atherosclerosis, adventitial fibrosis, and necrosis of the arterial wall.^2^, ^7^, ^11^ Although previously reported cases were typically accompanied by a pseudoaneurysm,^1^, ^2^, ^6^, ^7^, ^8^, ^9^, ^10^, ^11^ it was not noticeable on conventional angiography in the current case. Therefore, even with a massive epistaxis, it was challenging to immediately diagnose the patient with right ICA rupture based on only angiography results. However, considering the progress of follow‐up from the first visit, it was strongly suggested that epistaxis was caused by right ICA rupture.

Revascularization^1^, ^3^, ^4^, ^5^ and endovascular treatment^1^, ^2^, ^6^, ^7^, ^8^, ^9^, ^10^, ^11^ have been reported as treatments for the ICA rupture. Revascularization is curative and has the advantage that it can be performed even in cases with severe ICA stenosis, failed endovascular treatment, or BOT intolerance.^1^, ^3^ Contrastingly, it is technically dependent and has a low success rate, and there is a risk of decreased cerebral blood flow intraoperatively.^1^, ^3^ Endovascular treatment is minimally invasive, highly successful, and not technically dependent, whereas there is a risk of recurrence and intraoperative or postoperative thromboembolism.^1^, ^2^, ^11^ It should be noted that even passing the BOT, 5–22% of patients develop ischemic accidents postoperatively.^2^, ^6^

We selected revascularization using high‐flow bypass with trapping of the right ICA. Most authors have argued that endovascular treatment should be the first choice for ICA rupture with pseudoaneurysm in situations where patients could tolerate BOT.^1^, ^2^, ^6^, ^7^, ^8^, ^9^, ^10^, ^11^ The success rates for endovascular treatment and high‐flow bypass were 87% and 33%, respectively, according to a previous study.^1^ However, this case was a peculiar situation in which it was difficult to evaluate BOT. The patient had impaired consciousness at the time of the conventional angiography. In addition, indications for endovascular treatment, such as the existence of pseudoaneurysm or hemorrhage site on the conventional angiography with BOT intolerance, could not be identified. Consequently, revascularization was performed instead of endovascular treatment. In addition, revascularization greatly reduced the risk of hemorrhage from the right ICA during skull base reconstruction. Therefore, surgical manipulations could be performed safely.

This combination procedure in the current case had some limitations. High‐flow bypass with RA grafting is technically dependent, especially in emergencies.^1^, ^10^ Additionally, it is necessary to urgently assemble a cross‐departmental team of otolaryngologists and neurosurgeons: neurosurgeon‐otolaryngologist collaboration. Nevertheless, to rescue this type of case and obtain the best results, the combination procedure we performed would be a definitive curative treatment.

## 4 CONCLUSION

It is necessary to pay careful attention to the medical history of irradiation in patients with massive epistaxis and consider hemorrhage from ICA rupture as soon as possible. Our results suggest that simultaneous treatment of ICA rupture and osteoradionecrosis may provide satisfactory long‐term benefits.

## CONFLICT OF INTEREST

There is no conflict of interest to disclose.

## AUTHOR CONTRIBUTIONS

All authors made substantial contributions to the conception and design of the case report and interpretation of the data. All authors revised the manuscript, approved it to be published, and agreed to be accountable for all aspects of the work in ensuring that questions related to the accuracy or integrity of any part of the work are appropriately investigated and resolved. Each author participated in this work for an appropriate portion of the content. KT drafted the manuscript, and participated in skull base reconstruction and clinical revascularization. SG helped in drafting the manuscript. ST and JS participated in conventional angiography and designed the figure. KT participated in clinical revascularization.

## ETHICAL APPROVAL

Approval for this case report was obtained from the Ethics Committee of Japanese Red Cross Asahikawa Hospital (approval number: 202111–3). Written informed consent was obtained from the patient.

## Data Availability

The data that support the findings of this study are available from the corresponding author upon reasonable request.
